# Rapid and Accurate Ecotoxicological Assessment of Heavy Metals Using *Cyprinus carpio* Cells

**DOI:** 10.3390/life14091119

**Published:** 2024-09-05

**Authors:** Yun Haeng Lee, Myeong Uk Kuk, Ji Ho Park, Hojun Lee, Haneur Lee, Moon Kyoung So, Jee Hee Yoon, Yoo Jin Lee, Duyeol Kim, Byeonghyeon So, Minseon Kim, Jihae Park, Taejun Han, Joon Tae Park

**Affiliations:** 1 Division of Life Sciences, College of Life Sciences and Bioengineering, Incheon National University, Incheon 22012, Republic of Korea; yh.lee@inu.ac.kr (Y.H.L.); muk@inu.ac.kr (M.U.K.); 20248002@inu.ac.kr (J.H.P.); haneur.lee@ghent.ac.kr (H.L.); 202323088@inu.ac.kr (M.K.S.); yoojn0905@inu.ac.kr (J.H.Y.); juli9709@inu.ac.kr (Y.J.L.); papaya1130@inu.ac.kr (D.K.); tundra@inu.ac.kr (B.S.); alstjs0323@inu.ac.kr (M.K.); 2 Bio Environmental Science and Technology (BEST) Lab, Ghent University Global Campus, 119-5, Songdomunhwa-ro, Incheon 21985, Republic of Korea; hojun.lee@ugent.be; 3 Center for Environmental and Energy Research, Ghent University Global Campus, 119-5, Songdomunhwa-ro, Incheon 21985, Republic of Korea; jihae.park@ghent.ac.kr; 4 Department of Animal Sciences and Aquatic Ecology, Ghent University, Coupure Links 653-Block F, B-9000 Gent, Belgium; 5 Convergence Research Center for Insect Vectors, Incheon National University, Incheon 22012, Republic of Korea

**Keywords:** rapid ecotoxicological assessment, accurate ecotoxicological assessment, *Cyprinus carpio*

## Abstract

Heavy metals have serious negative effects on various aquatic organisms, and therefore rapid and accurate ecotoxicological assessments of heavy metals are necessary. Fish-derived cells sensitive to heavy metals have been used as valuable tools for ecotoxicological assessments. However, this method requires a minimum toxicity treatment time of 96 h, which limits its use when rapid ecotoxicological assessments are required or ecotoxicological assessments of a large number of toxicants are performed. In this study, these limitations were overcome by adjusting parameters including the concentration of fetal bovine serum (FBS) in the medium and the treatment time of the toxicant. Specifically, we found that the maximum time for fish cells to remain unstarved was 6 h when using a medium containing 1% FBS. We applied both parameters to the ecotoxicological assessment (using a medium containing 1% FBS for the toxicity assessment and treating the toxicant for only 6 h). Surprisingly, these adjusted parameters allowed us to obtain faster and more accurate data than the traditional assessment. This improvement was due to the new assessment conditions that minimized the possibility that the growth-inducing effects of nutrients present in excess in the medium could interfere with the cellular response to the toxicant. The accuracy of this assessment was not limited to measuring the toxicity of heavy metals. In conclusion, we have established an ecotoxicity assessment that can generate rapid and accurate data on heavy metals. This new platform will become the cornerstone of rapid and accurate ecotoxicity assessments of heavy metals.

## 1. Introduction

Heavy metals have significant negative effects on various aquatic plants and animals. Continuous exposure to heavy metals gradually increases the concentration of toxic substances in the body of aquatic organisms. The toxic substances accumulated in the body can easily affect larvae and embryos, causing spinal deformation, morphological changes, decreased cardiac activity, and increased mortality. Heavy metals not only have negative effects on aquatic organisms but can also affect humans who consume aquatic organisms. Consuming aquatic organisms contaminated with heavy metals can cause various health problems, such as organ damage, cancer, and developmental disorders. Aquatic environments are subject to contamination by a variety of heavy metals, hence effective monitoring techniques must be developed to protect the integrity of aquatic environments [1].

Fish exhibit rapid physiological responses to pollutants and are highly sensitive to changes in water quality, allowing them to identify potential hazards posed by pollutants [2]. Fish-based ecotoxicological assessments have been used as a cornerstone for water quality improvement, but they also have limitations [3]. Fish respond not only to pollutants but also to a variety of stressors. The wide range of reactions makes data evaluation more difficult and can lead to incorrect assessments of toxicity [4]. Considering these findings, the sole use of fish-based ecotoxicology assessments is questionable. Therefore, more effective means that can be used alone or in conjunction with fish-based ecotoxicological assessments are needed. As an alternative to ecotoxicological assessment using fish, a method using fish-derived cell lines has been used. Fish-derived cells provide a more consistent testing environment by eliminating variations resulting from different behaviors and responses [5]. For example, the rainbow trout-derived fish cell line RTG-2 has been used for ecotoxicological evaluation of several toxicants [6,7]. Additionally, the cell line PLHC-1 derived from *Poeciliopsis lucida* was the first to demonstrate the carcinogenicity of 7,12-dimethylbenanthracene [8].

The period of ecotoxicity evaluation using fish cells varies depending on the purpose (approximately 4 to 21 days) [9,10]. However, the long duration of ecotoxicological assessments limits the number of toxicants that can be performed within a given period of time. In addition, when toxicity evaluation of toxicants needs to be performed quickly, the toxicity evaluation system currently in use cannot meet these requirements. There is an urgent need to establish a platform that can perform ecotoxicity assessments in a short period of time.

When performing ecotoxicological assessments using fish cells, no clear standard has been established for the concentration of fetal bovine serum (FBS) in the medium [11,12]. Using media containing a high percentage of FBS allows the response of fish cells to toxicants to be counteracted by the proliferation-inducing effect of the nutrients present in the media [12,13]. Alternatively, using media containing a low percentage of FBS starves fish cells from nutrient depletion and prevents them from responding appropriately to toxicants [12,13]. Therefore, determining the optimal period during which cells do not reach starvation even when cells are cultured in media with a low percentage of FBS will be an important standard for ecotoxicity assessment.

*Cyprinus carpio* (*C. carpio*) is predominantly found in East Asia, especially Korea and Japan [14]. This fish is an ecologically important species that inhabits clear rivers and is a central member of the river ecosystem, engaging in complex interactions within the ecosystem [14]. In addition, *C. carpio* serves as a key indicator for environmental toxicity assessment due to its early response to adverse effects resulting from water pollution [15].

In this study, it was developed a platform for the ecotoxicity assessment of heavy metals using *C. carpio* cells. This platform enables rapid and accurate ecotoxicity assessment of heavy metals compared to conventional ecotoxicity assessments. Furthermore, the use of this assessment platform is not limited to heavy metals. Here, it was proposed a new ecotoxicity assessment platform that can produce accurate ecotoxicity results very quickly.

## 2. Materials and Methods

### 2.1. C. carpio Cells

*C. carpio* cells were established from *C. carpio* fish according to the previous study and exhibited fibroblast morphology consistent with the previous study [16]. Specifically, *C. carpio* was collected from riverine habitats using a specific size criterion of 3–5 cm. Primary cell cultures were started by aseptically collecting tissue from *C. carpio*. Tissues were minced for 5 min using a scalpel blade (No. 11; S2771, Sigma Aldrich, St. Louis, MO, USA). Dulbecco’s modified Eagle’s medium containing 25 mM glucose and supplemented with 15% fetal bovine serum (FBS) (SH30919.03; Hyclone, Waltham, MA, USA), 100 U/mL penicillin, and 100 μg/mL streptomycin (SV30079.01; Hyclone) was used for primary cell culture. The minced tissues were placed in 50 mL conical tubes containing 10 mL cell culture medium supplemented with 2 mg/mL collagenase A (10103578001; Sigma Aldrich). Then, they were shaken at 180× *g* for 1 h at 37 °C and were filtered through a 100 μm sieve (93100; SPL, Pocheon-si, Gyeonggi-do, Republic of Korea). The filtrates were collected and centrifuged at 650× *g* for 2 min to obtain pellets. The pellets were washed twice with 15 mL of phosphate-buffered saline (PBS; 21-031-CVC; Corning, Corning, NY, USA). The washed pellets were suspended in 15 mL of culture medium and plated on cell plates. The cells were then cultured at 37 °C in an ambient atmosphere containing 20% O_2_ and 5% CO_2_. Cell viability was assessed using the Cedex HiRes Analyser (05650216001; Roche, Basel, Switzerland).

### 2.2. Ecotoxicological Assessment

For the ecotoxicological assessment, cells were seeded in a 96-well plate, with 3000 cells in each well in medium supplemented with 15% or 1% FBS. Subsequently, the cells were exposed to varying concentrations of toxicants, CuSO_4_ (C2284-25ML; Sigma Aldrich, St. Louis, MO, USA), SDS (L3771; Sigma Aldrich), titanium (366994; Sigma Aldrich), titanium nitride (595063; Sigma Aldrich), metolachlor (36163; Sigma Aldrich), linuron (36141; Sigma Aldrich), 2,4,6-trichlorophenol (T55301; Sigma Aldrich), and perfluorononanoic acid (394459; Sigma Aldrich). Details of the toxicant concentrations used in the ecotoxicological assessment are described in Table 1.

The determination of cell numbers was carried out using a DNA content-based approach [17]. Specifically, cells were lysed in 50 μL of 0.2% SDS (436143; Sigma Aldrich) after being twice rinsed with PBS (21-031-CVC; Corning). For 2 h, the plates were incubated at 37 °C. Then, wells were filled with 150 μL of SYBR Green I nucleic acid gel stain (1:1000 in DW; S-7567; Molecular Probes, Eugene, OR, USA). Fluorescence intensity was measured using a fluorescence microplate reader (Infinite 200 PRO) to assess the number of cells. For every experimental group, the mean and standard deviation from three samples were calculated. To evaluate cell viability, the value at each concentration was divided by the value from 0 ppm. All data presented were performed in biological triplicates using three samples in each experiment.

### 2.3. Statistical Analyses

The calculation of parameters such as the no observed effect concentration (NOEC), effective concentration to induce 10% maximal responses (EC_10_), a semi-effective concentration (EC_50_) value, and 95% confidence limits (CI) was performed using GraphPad Prism 7.0 (Boston, MA, USA).

## 3. Results

### 3.1. Establishment of Ecotoxicological Evaluation Conditions Using C. carpio Cells

To establish the criteria for FBS concentration in media for ecotoxicological evaluation using fish cells, changes in cell number were investigated using media containing 15% FBS, the most commonly used FBS concentration in fish cell culture [18,19,20], and media containing 1% FBS, the lowest FBS concentration [21].

**Figure 1:**
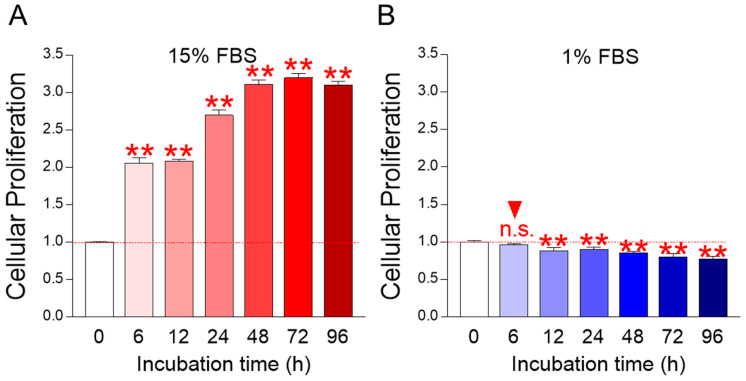
Establishment of ecotoxicological evaluation conditions using *Cyprinus carpio* (*C. carpio;* English name: Common carp) cells. (**A**) Measurement of cell proliferation of *C. carpio* cells in medium containing 15% FBS. Cells showed rapid cell proliferation starting at 6 h and plateaued proliferation starting at 48 h. ** *p* < 0.01, Student’s *t*-test. Mean ± S.D., *n* = 6. Raw data for cellular proliferation was provided in Table S1. (**B**) Measurement of cell proliferation of *C. carpio* cells in medium containing 1% FBS. Cells showed no difference in cell number until 6 h, but gradual decrease starting at 12 h. n.s. = not significant, ** *p* < 0.01, Student’s *t*-test. Mean ± S.D., *n* = 6. Raw data for cellular proliferation was provided in Table S2.

*C. carpio* cells in a medium containing 15% FBS showed rapid cell proliferation starting at 6 h and plateaued proliferation starting at 48 h (Figure 1A). These data indicate that using media containing 15% FBS may not provide accurate ecotoxicological data during the initial 6–48 h due to the growth-inducing effects of nutrients present in the media.

*C. carpio* cells in a medium containing 1% FBS showed no difference in cell number until 6 h, but gradual decrease starting at 12 h (Figure 1B). These data suggest that *C. carpio* cells did not reach starvation when cultured for 6 h in a medium containing 1% FBS.

Taken together, these data indicate that cellular responses to toxicants can be accurately measured if ecotoxicological assessments can be completed in up to 6 h in media containing 1% FBS.

### 3.2. Schematic Diagram of Ecotoxicological Evaluation of Traditional and New Methods

**Figure 2:**
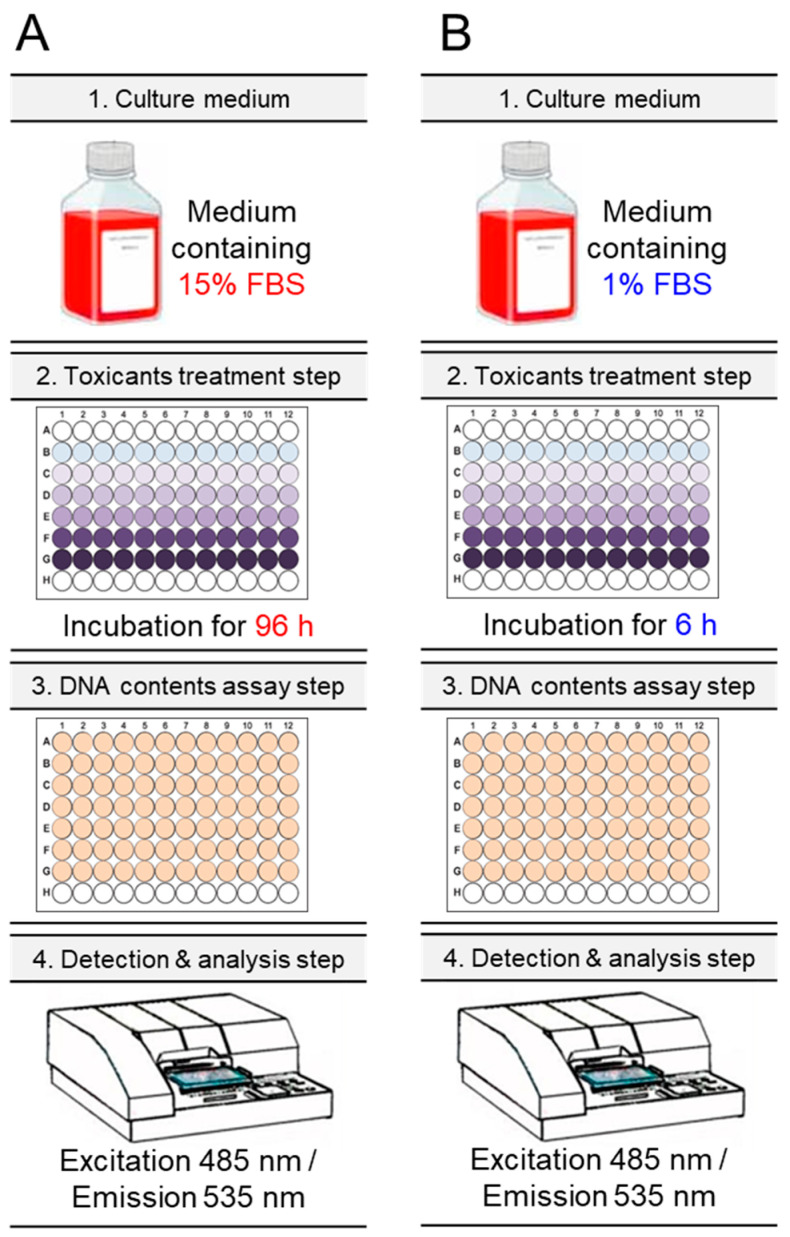
Schematic diagram of ecotoxicological evaluation of traditional and new methods. (**A**) *C. carpio* cells were exposed to various concentrations of toxicants for 96 h using medium containing 15% FBS. Cell numbers were decided using a DNA content-based approach [17]. (**B**) *C. carpio* cells were exposed to various concentrations of toxicants for 6 h using medium containing 1% FBS. Cell numbers were decided using a DNA content-based approach [17].

The establishment of new criteria allowed comparison of ecotoxicological assessments using these conditions with traditional methods. In traditional ecotoxicological assessments, *C. carpio* cells were exposed to toxicants for 96 h using a medium containing 15% FBS [18,19,20] (Figure 2A). In a new ecotoxicological assessment, new parameters (using a medium containing 1% FBS and treating the toxicant for only 6 h) were applied to *C. carpio* cells (Figure 2B). Cell numbers were quantified by a DNA-based method using a fluorometer with excitation of 485 nm and emission of 535 nm [17] (Figure 2A,B).

### 3.3. Comparison of Traditional and New Methods for the Ecotoxicity of CuSO_4_ Using C. carpio Cells

**Figure 3:**
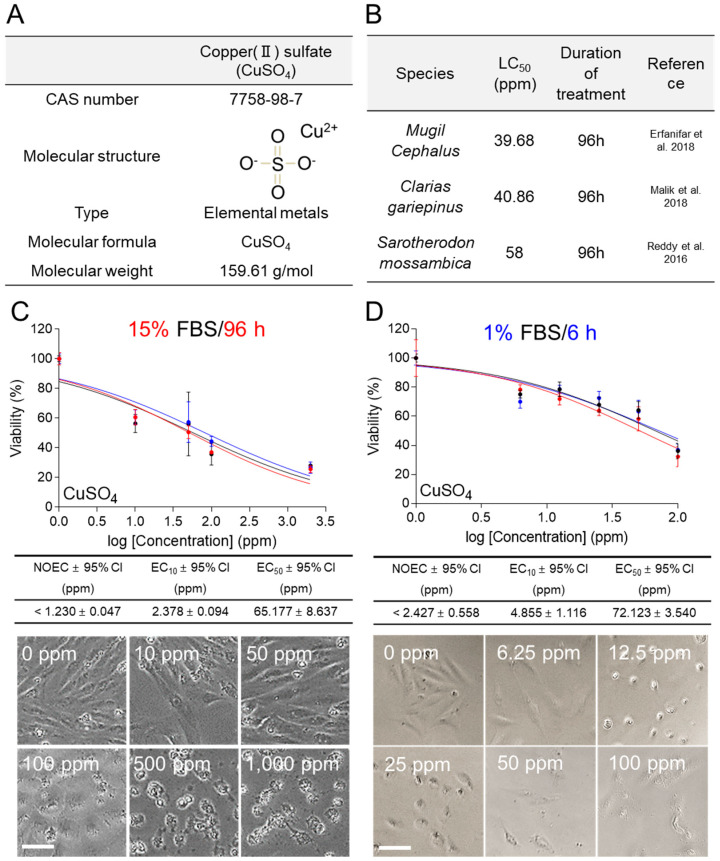
Comparison of traditional and new methods for the ecotoxicity of CuSO_4_ using *C. carpio* cells. (**A**) The CAS numbers, molecular structures, types, molecular formulas, and molecular weights for CuSO_4_ were indicated. (**B**) LC_50_ values in *Mugil Cephalus* (English name: Flathead grey mullet) [23], *Clarias gariepinus* (English name: North African catfish) [24], and *Sarotherodon mossambica* (English name: Mozambique tilapia) [25] were provided for reference. (**C**) In ecotoxicological assessment using medium containing 15% FBS, cell viability was assessed after treating *C. carpio* cells with different concentrations of CuSO_4_ (0, 10, 50, 100, and 2000 ppm) for 96 h. No observed effect concentration (NOEC), effective concentration to induce 10% maximal responses (EC_10_), EC_50_ values, and 95% confidence intervals (95% CI) were calculated using GraphPad Prism 7.0. All data presented were performed in biological triplicates using three samples in each experiment. Representative images at indicated concentrations were shown. Scale bar 10 μm. Raw data for ecotoxicological assessment was provided in Table S3. (**D**) In ecotoxicological assessment using medium containing 1% FBS, cell viability was assessed after treating *C. carpio* cells with different concentrations of CuSO_4_ (0, 6.25, 12.5, 25, 50, and 100 ppm) for 6 h. NOEC, EC_10_, EC_50_, and 95% CI were calculated using GraphPad Prism 7.0. All data presented were performed in biological triplicates using three samples in each experiment. Representative images at indicated concentrations were shown. Scale bar 10 μm. Raw data for ecotoxicological assessment was provided in Table S4.

Copper, particularly in the form of copper(II) sulfate (CuSO_4_), is of importance due to its relatively persistent presence in the environment [22]. CuSO_4_ (CAS number 7758-98-7; molecular weight 159.61 g/mol) is a metal used in industrial processes and pesticides (Figure 3A). The lethal concentration 50 (LC_50_) obtained from ecotoxicological assessment using fish species such as *Mugil Cephalus* (96 h toxicant treatment) [23], *Clarias gariepinus* (96 h toxicant treatment) [24], and *Sarotherodon mossambica* (96 h toxicant treatment) [25] were 39.68, 40.86, and 58 ppm, respectively (Figure 3B).

In ecotoxicological assessment using medium containing 15% FBS, *C. carpio* cells were exposed to different concentrations of CuSO_4_ (0, 10, 50, 100, and 1000 ppm) for 96 h. CuSO_4_ exhibited an EC_50_ value of 65.117 ± 8.637 (mean ± 95% CI) ppm and its effect on cell viability was observed in a concentration-dependent manner (Figure 3C). The concentration-dependent decrease in cell viability is indicative of the toxic effects attributed to CuSO_4_ (Figure 3C). The NOEC and EC_10_ values were less than 1.230 ± 0.047 (mean ± 95% CI) ppm and 2.378 ± 0.094 (mean ± 95% CI) ppm, respectively, highlighting the highly toxic nature of CuSO_4_ even at extremely low concentrations (Figure 3C). From 100 ppm concentration, morphological changes were clearly observed (Figure 3C).

In ecotoxicological assessment using medium containing 1% FBS, *C. carpio* cells were exposed to different concentrations of CuSO_4_ (0, 6.25, 12.5, 25, 50, and 100 ppm) for 6 h. CuSO_4_ exhibited an EC_50_ value of 72.123 ± 3.540 (mean ± 95% CI) ppm and its effect on cell viability was observed in a concentration-dependent manner (Figure 3D). The NOEC and EC_10_ values were less than 2.427 ± 0.058 (mean ± 95% CI) ppm and 4.855 ± 1.116 (mean ± 95% CI) ppm, respectively (Figure 3D). Even at 12.5 ppm, morphological changes were clearly observed (Figure 3D).

The EC_50_ values for CuSO_4_ in the 15% FBS/96 h (65.117 ± 8.637 ppm) and 1% FBS/6 h (72.123 ± 3.540 ppm) groups were both close to the LC_50_ values (39.68, 40.86, and 58 ppm) measured using other fish species (Figure 3C,D).

### 3.4. Comparison of Traditional and New Methods for the Ecotoxicity of SDS Using C. carpio Cells

**Figure 4:**
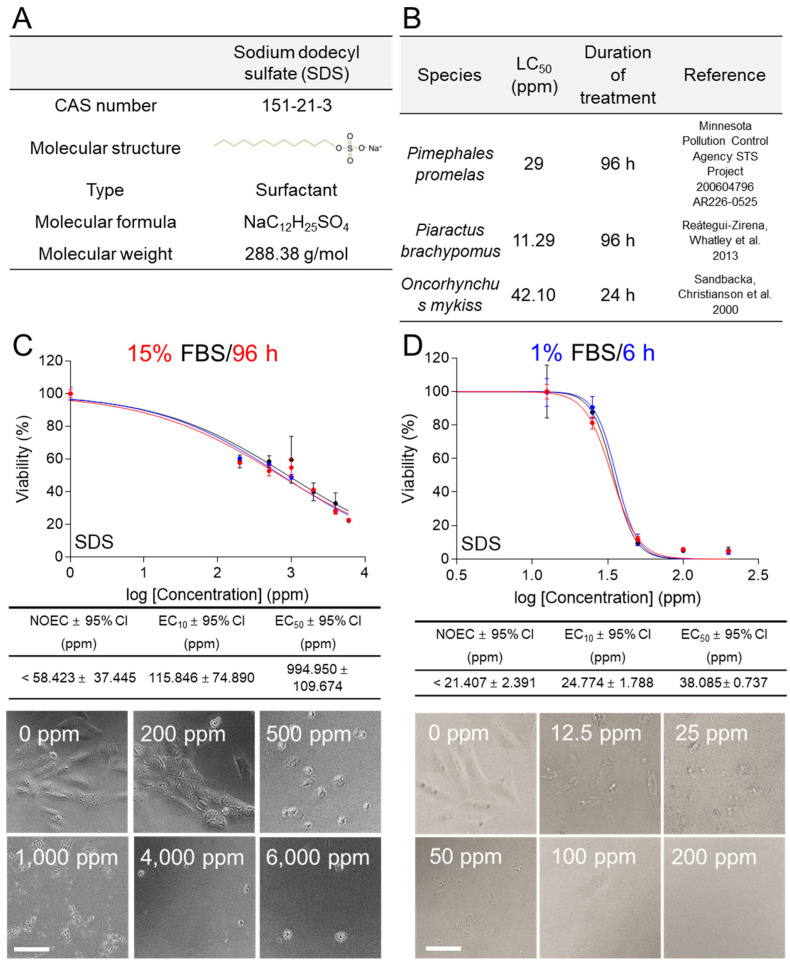
Comparison of traditional and new methods for the ecotoxicity of SDS using *C. carpio* cells. (**A**) The CAS numbers, molecular structures, types, molecular formulas, and molecular weights for SDS were indicated. (**B**) LC_50_ values in *Pimephales promelas* (English name: Fathead minnow; Minnesota Pollution Control Agency STS Project 200604796 AR226-0525), *Piaractus brachypomus* (English name: Pirapitinga) [27], and *Oncorhynchus mykiss* (English name: Rainbow trout) [28] were provided for reference. (**C**) In ecotoxicological assessment using medium containing 15% FBS, cell viability was assessed after treating *C. carpio* cells with different concentrations of SDS (0, 200, 500, 1000, 2000, 4000, and 6000 ppm) for 96 h. NOEC, EC_10_, EC_50_, and 95% CI were calculated using GraphPad Prism 7.0. All data presented were performed in biological triplicates using three samples in each experiment. Representative images at indicated concentrations were shown. Scale bar 10 μm. Raw data for ecotoxicological assessment were provided in Table S5. (**D**) In ecotoxicological assessment using medium containing 1% FBS, cell viability was assessed after treating *C. carpio* cells with different concentrations of SDS (0, 12.5, 25, 50, 100, and 200 ppm) for 6 h. NOEC, EC_10_, EC_50_, and 95% CI were calculated using GraphPad Prism 7.0. All data presented were performed in biological triplicates using three samples in each experiment. Representative images at indicated concentrations were shown. Scale bar 10 μm. Raw data for ecotoxicological assessment were provided in Table S6.

To determine whether traditional and new methods can be applied to the ecotoxicity assessment of toxicants other than heavy metals, an evaluation was performed using sodium dodecyl sulfate (SDS), which is used as a surfactant. SDS, which has a CAS number of 151-21-3 and a molecular weight of 288.38 g/mol, is used in many cleaning and hygiene products as an anionic surfactant [26] (Figure 4A). The LC_50_ obtained from ecotoxicological assessment using fish species such as *Pimephales promelas* (96 h toxicant treatment) (Minnesota Pollution Control Agency STS Project 200604796 AR226-0525), *Piaractus brachypomus* (96 h toxicant treatment) [27], and *Oncorhynchus mykiss* (24 h toxicant treatment) [28] were 29, 11.29, and 42.10 ppm, respectively (Figure 4B).

In ecotoxicological assessment using medium containing 15% FBS, *C. carpio* cells were exposed to different concentrations of SDS (0, 200, 500, 1000, 2000, 4000, and 6000 ppm) for 96 h. In the group, SDS exhibited an EC_50_ value of 994.950 ± 109.674 (mean ± 95% CI) ppm (Figure 4C). The NOEC and EC_10_ values were less than 58.423 ± 37.445 ppm and 115.846 ± 74.890 (mean ± 95% CI) ppm, respectively, indicating a lack of toxicity at low concentrations (Figure 4C). From 500 ppm concentration, morphological changes were clearly observed (Figure 4C).

In ecotoxicological assessment using medium containing 1% FBS, *C. carpio* cells were exposed to different concentrations of SDS (0, 12.5, 25, 50, 100, and 200 ppm) for 6 h. SDS exhibited an EC_50_ value of 38.085 ± 0.737 (mean ± 95% CI) ppm and its effect on cell viability was observed in a sigmoidal manner (Figure 4D). The NOEC and EC_10_ values were less than 21.407 ± 2.391 (mean ± 95% CI) ppm and 24.774 ± 1.788 (mean ± 95% CI) ppm, respectively (Figure 4D). Even at 25 ppm, morphological changes were clearly observed (Figure 4D). The EC_50_ for SDS in the 1% FBS/6 h group (38.085 ± 0.737 ppm) was within the range of LC_50_ values measured using other fish.

The EC_50_ for SDS in the 1% FBS/6 h group (38.085 ± 0.737 ppm) was closer to the LC_50_ values (29, 11.29, and 42.10 ppm) measured using other fish species than the 15% FBS/96 h (994.950 ± 109.674) group (Figure 4C,D).

### 3.5. Ecotoxicological Assessment of Heavy Metals Using the New Method

**Figure 5:**
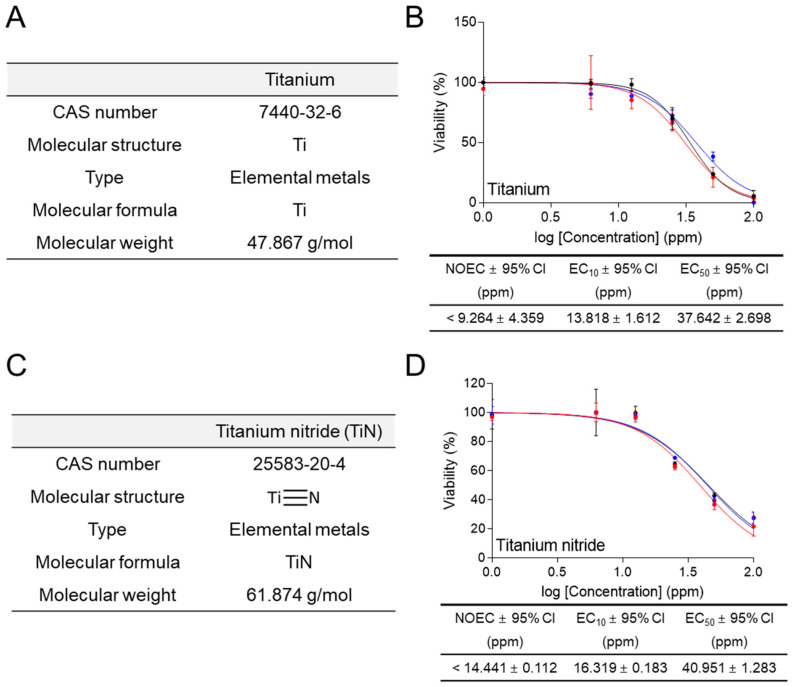
Ecotoxicological assessment of heavy metals using the new method. (**A**) The CAS numbers, molecular structures, types, molecular formulas, and molecular weights for titanium were indicated. (**B**) In ecotoxicological assessment using medium containing 1% FBS, cell viability was assessed after treating *C. carpio* cells with different concentrations of titanium (0, 6.25, 12.5, 25, 50, and 100 ppm) for 6 h. NOEC, EC_10_, EC_50_, and 95% CI were calculated using GraphPad Prism 7.0. All data presented were performed in biological triplicates using three samples in each experiment. Raw data for ecotoxicological assessment were provided in Table S7. (**C**) The CAS numbers, molecular structures, types, molecular formulas, and molecular weights for titanium nitride were indicated. (**D**) In ecotoxicological assessment using medium containing 1% FBS, cell viability was assessed after treating *C. carpio* cells with different concentrations of titanium nitride (0, 6.25, 12.5, 25, 50, and 100 ppm) for 6 h. NOEC, EC_10_, EC_50_, and 95% CI were calculated using GraphPad Prism 7.0. All data presented were performed in biological triplicates using three samples in each experiment. Raw data for ecotoxicological assessment were provided in Table S8.

The establishment of a rapid and accurate ecotoxicity assessment method has led to the investigation of the ecotoxicity of various heavy metals other than CuSO_4_. Titanium (CAS number 7440-32-6; molecular weight 159.61 g/mol) is a metal used to alloy many metals, including aluminum, molybdenum, and iron (Figure 5A). Moreover, it is widely used as a pigment in household paints, artist paints, plastics, and enamels. Titanium can affect lung function and cause lung diseases such as pleural effusion, and chest pain with tightness and shortness of breath [29,30]. In ecotoxicological assessment using medium containing 1% FBS, *C. carpio* cells were exposed to different concentrations of titanium (0, 6.25, 12.5, 25, 50, and 100 ppm) for 6 h. Titanium exhibited an EC_50_ value of 37.642 ± 2.698 (mean ± 95% CI) ppm and its effect on cell viability was observed in a sigmoidal manner (Figure 5B). The NOEC and EC_10_ values were less than 9.264 ± 4.359 (mean ± 95% CI) ppm and 13.818 ± 1.612 (mean ± 95% CI) ppm, respectively (Figure 5B).

Titanium nitride (Tin) (CAS number 25583-20-4; molecular weight 61.874 g/mol) is a metal used to alloy with many metals, including aluminum, molybdenum, and iron (Figure 5C). As the concentration and exposure period of TiN increased, the mortality and malformation of zebrafish embryos gradually increased [31]. Specifically, the body length was shortened and there was a notable decrease in the rate of hatching and motility. In addition, TiN impacts the development of the liver, heart, and nerves by raising the level of reactive oxygen species and lowering the antioxidant potential [31]. In ecotoxicological assessment using medium containing 1% FBS, *C. carpio* cells were exposed to different concentrations of TiN (0, 6.25, 12.5, 25, 50, and 100 ppm) for 6 h. TiN exhibited an EC_50_ value of 40.951 ± 1.283 (mean ± 95% CI) ppm and its effect on cell viability was observed in a sigmoidal manner (Figure 5D). The NOEC and EC_10_ values were less than 14.441 ± 0.112 (mean ± 95% CI) ppm and 16.319 ± 0.183 (mean ± 95% CI) ppm, respectively (Figure 5D).

### 3.6. Ecotoxicological Assessment of Pesticide Using the New Method

**Figure 6:**
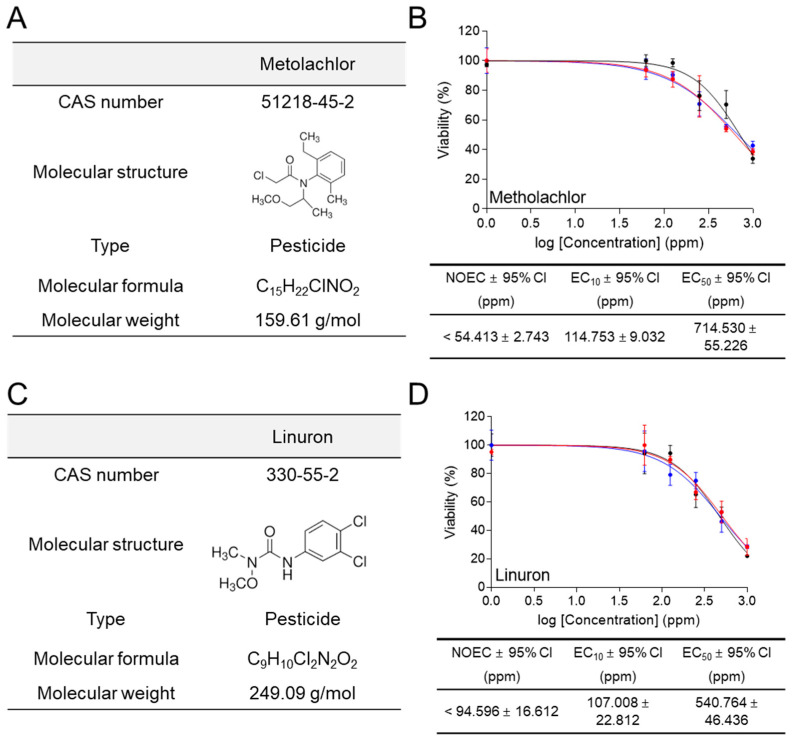
Ecotoxicological assessment of pesticide using the new method. (**A**) The CAS numbers, molecular structures, types, molecular formulas, and molecular weights for metolachlor were indicated. (**B**) In ecotoxicological assessment using medium containing 1% FBS, cell viability was assessed after treating *C. carpio* cells with different concentrations of metolachlor (0, 6.25, 12.5, 25, 50, and 100 ppm) for 6 h. NOEC, EC_10_, EC_50_, and 95% CI were calculated using GraphPad Prism 7.0. All data presented were performed in biological triplicates using three samples in each experiment. Raw data for ecotoxicological assessment were provided in Table S9. (**C**) The CAS numbers, molecular structures, types, molecular formulas, and molecular weights for linuron were indicated. (**D**) In ecotoxicological assessment using medium containing 1% FBS, cell viability was assessed after treating *C. carpio* cells with different concentrations of linuron (0, 6.25, 12.5, 25, 50, and 100 ppm) for 6 h. NOEC, EC_10_, EC_50_, and 95% CI were calculated using GraphPad Prism 7.0. All data presented were performed in biological triplicates using three samples in each experiment. Raw data for ecotoxicological assessment were provided in Table S10.

Metolachlor (CAS number 51218-45-2; molecular weight 159.61 g/mol) is a pesticide group (Figure 6A). Metolachlor is a selective herbicide that controls weeds by inhibiting the synthesis of long-chain fatty acids [32]. Metolachlor has been shown to be genotoxic to human lymphocytes and fish, negatively affecting growth and development [33,34]. In ecotoxicological assessment using a medium containing 1% FBS, *C. carpio* cells were exposed to different concentrations of metolachlor (0, 62.5, 125, 250, 500, and 1000 ppm) for 6 h. Metolachlor exhibited an EC_50_ value of 714.530 ± 55.226 (mean ± 95% CI) ppm, and its effect on cell viability was rapidly observed starting at 500 ppm (Figure 6B). The NOEC and EC_10_ values were less than 54.413 ± 2.743 (mean ± 95% CI) ppm and 114.753 ± 9.032 (mean ± 95% CI) ppm, respectively (Figure 6B).

Linuron (CAS number 330-55-2; molecular weight 249.09 g/mol) is a pesticide group (Figure 6C). Linuron is a phenylurea herbicide used to control the growth of target weed plants by inhibiting photosynthesis [35]. Linuron is considered an endocrine disruptor because it acts as an androgen receptor antagonist and can cause reproductive harm in animals [36]. In ecotoxicological assessment using a medium containing 1% FBS, *C. carpio* cells were exposed to different concentrations of metolachlor (0, 62.5, 125, 250, 500, and 1000 ppm) for 6 h. Linuron exhibited an EC_50_ value of 540.764 ± 46.436 (mean ± 95% CI) ppm, and its effect on cell viability was rapidly observed starting at 500 ppm (Figure 6D). The NOEC and EC_10_ values were less than 94.596 ± 16.612 (mean ± 95% CI) ppm and 107.008 ± 22.812 (mean ± 95% CI) ppm, respectively (Figure 6D).

### 3.7. Ecotoxicological Assessment of Industrial Waste Using the New Method

**Figure 7:**
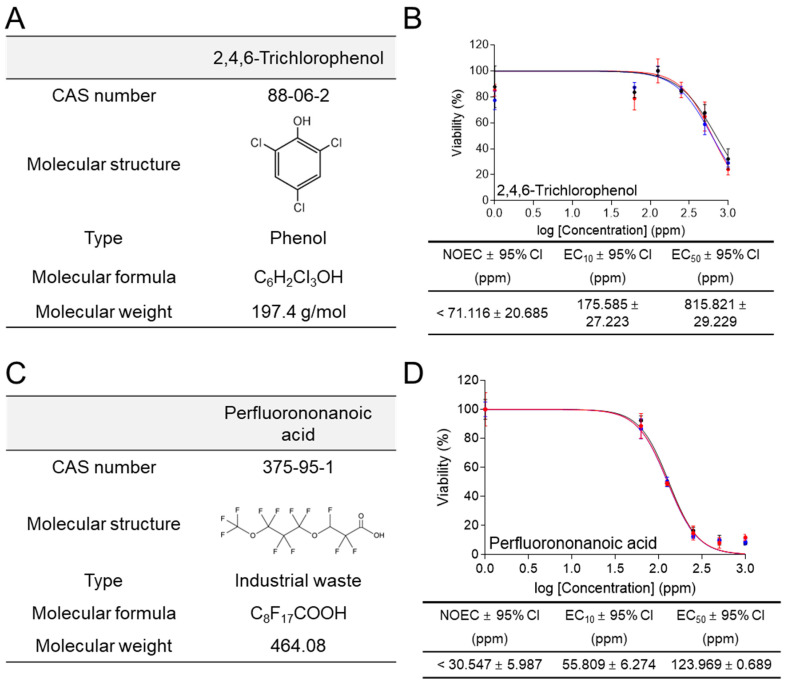
Ecotoxicological assessment of industrial waste using the new method. (**A**) The CAS numbers, molecular structures, types, molecular formulas, and molecular weights for 2,4,6-trichlorophenol were indicated. (**B**) In ecotoxicological assessment using medium containing 1% FBS, cell viability was assessed after treating *C. carpio* cells with different concentrations of 2,4,6-trichlorophenol (0, 6.25, 12.5, 25, 50, and 100 ppm) for 6 h. NOEC, EC_10_, EC_50_, and 95% CI were calculated using GraphPad Prism 7.0. All data presented were performed in biological triplicates using three samples in each experiment. Raw data for ecotoxicological assessment were provided in Table S11. (**C**) The CAS numbers, molecular structures, types, molecular formulas, and molecular weights for perfluorononanoic acid were indicated. (**D**) In ecotoxicological assessment using medium containing 1% FBS, cell viability was assessed after treating *C. carpio* cells with different concentrations of perfluorononanoic acid (0, 6.25, 12.5, 25, 50, and 100 ppm) for 6 h. NOEC, EC_10_, EC_50_, and 95% CI were calculated using GraphPad Prism 7.0. All data presented were performed in biological triplicates using three samples in each experiment. Raw data for ecotoxicological assessment were provided in Table S12.

2,4,6-trichlorophenol (CAS number 88-06-2; molecular weight 197.4 g/mol) is an industrial waste group (Figure 7A). 2,4,6-trichlorophenol is produced through the breakdown of other chemicals present in industrial wastewater [37]. Oral ingestion of 2,4,6-trichlorophenol has been shown to cause lymphoma, leukemia, and liver cancer in animals [38]. In ecotoxicological assessment using a medium containing 1% FBS, *C. carpio* cells were exposed to different concentrations of metolachlor (0, 62.5, 125, 250, 500, and 1000 ppm) for 6 h. 2,4,6-trichlorophenol exhibited an EC_50_ value of 815.821 ± 29.229 (mean ± 95% CI) ppm, and its effect on cell viability was rapidly observed starting at 500 ppm (Figure 7B). The NOEC and EC_10_ values were less than 71.116 ± 20.685 (mean ± 95% CI) ppm and 175.585 ± 27.223 (mean ± 95% CI) ppm, respectively (Figure 7B).

Perfluorononanoic acid (CAS number 375-95-1; molecular weight 464.08 g/mol) is an industrial waste group (Figure 7C). Perfluorononanoic acid is widely used in the automotive, construction, and electronics industries as it is used to produce stain-resistant and chemically inert coatings [39]. Perfluorononanoic acid has been classified as a toxic substance of very high concern due to reproductive toxicity, and its manufacture and use are strictly restricted [40]. In ecotoxicological assessment using a medium containing 1% FBS, *C. carpio* cells were exposed to different concentrations of metolachlor (0, 62.5, 125, 250, 500, and 1000 ppm) for 6 h. Linuron exhibited an EC_50_ value of 123.969 ± 0.689 (mean ± 95% CI) ppm, and its effect on cell viability was rapidly observed starting at 125 ppm (Figure 7D). The NOEC and EC_10_ values were less than 30.547 ± 5.987 (mean ± 95% CI) ppm and 55.809 ± 6.274 (mean ± 95% CI) ppm, respectively (Figure 7D).

## 4. Discussion

Heavy metals flowing from factories and farms near rivers pose a threat to river ecosystems [41,42]. Within the ecosystem, fish play an important role in maintaining the ecological balance and serve as sentinel organisms for pollution detection [43]. Fish are sensitive to changes in water quality and show identifiable physiological responses to pollutants [2,44]. The presence or absence of specific fish populations serves as an indicator of changes in water quality [2,44]. Fish-based ecotoxicological assessments provide a multidimensional view of the ecosystem and are a useful tool for assessing water quality [45]. Despite their value, fish-based assessments have inherent limitations. They require the sacrifice of live fish for each assessment, which raises ethical concerns [3]. Furthermore, the variable responses of fish to different stressors can lead to inconsistent results and potentially inaccurate assessments of water quality [4]. To address these shortcomings, fish cell line-based ecotoxicological assessments have been used. Fish-derived cell lines provide a consistent testing environment by reducing the variability associated with individual fish behavior [46]. Furthermore, fish cells exhibit enhanced sensitivity to toxicants and respond rapidly to low concentrations of toxicants [46]. Therefore, ecotoxicity assessment using fish cells is being used as an alternative to ecotoxicity assessment using fish [47,48]. However, the ecotoxicity assessment method using fish cells had room for improvement in two aspects. First, since this assessment method requires at least 96 h of toxicity treatment [9,10], it cannot be used in cases where rapid ecotoxicity assessment is required. Second, this assessment method showed discrepancies from the ecotoxicity assessment data using fish, so there was room for improvement in terms of accuracy [49,50]. Therefore, much effort has been made to improve the measurement speed and accuracy by adjusting the parameters. In this study, the minimum time before cells did not reach starvation was determined when the minimum concentration of FBS was used. Specifically, when the medium containing 1% FBS was used, the minimum time before cells did not reach starvation was 6 h. Surprisingly, under these conditions, the fish cells effectively responded to the toxicant. Furthermore, ecotoxicological data obtained from the 1% FBS/6 h group were similar or more accurate than those obtained from the 15% FBS/96 h group. We propose that this new ecotoxicological assessment platform, capable of generating rapid and accurate data, would be an essential tool for environmental monitoring and protection. However, we acknowledge that further research is needed because the conditions were set using only 1% or 15% FBS concentration. If conditions are set using various FBS concentrations, it is expected that better ecotoxicity assessment conditions can be set.

The number of chemicals registered for commercial production is estimated at approximately 350,000, and new chemicals are constantly being produced [51]. Since only a small number of these chemicals have been evaluated for ecotoxicity, the remaining chemicals should be evaluated as soon as possible [52]. Traditional ecotoxicity assessment using fish cells takes a long time to complete due to the time required to treat the toxicants. It has been difficult to meet the demand for ecotoxicity assessment of a large number of toxicants with traditional methods. Therefore, a new ecotoxicity assessment system that can meet this demand is needed. The ecotoxicity assessment established in this study can sufficiently meet this demand. For example, one researcher can complete an ecotoxicity assessment of 15 to 20 toxicants per day. In addition, by starting a second set of tests before completing the first test, one researcher can double the number of toxicants evaluated in a given time. Therefore, this innovative ecotoxicity assessment platform can collect large-scale ecotoxicity data in a short period of time. We believe that this novel ecotoxicity assessment platform, capable of generating rapid and comprehensive data, will be an essential tool for enabling ecotoxicity assessments of numerous toxicants.

Industrial wastewater, even in relatively small quantities, accumulates in the food chain and threatens aquatic ecosystems [53]. It contains various hazardous substances such as heavy metals, industrial chemicals, pesticides, pharmaceuticals, and surfactants [54]. Therefore, it is essential to develop a universal ecotoxicity assessment that can evaluate the ecotoxicity of toxicants. In this study, we investigated whether the developed system can be applied to the ecotoxicity assessment of toxicants other than heavy metals. SDS, an anionic surfactant used in many cleaning and hygiene products, was selected as the toxicant. The newly developed method provided more accurate ecotoxicity data on SDS than the conventional 96 h method. Moreover, metolachlor (pesticide), linuron (pesticide), 2,4,6-trichlorophenol (industrial wastewater), and perfluorononanoic acid (industrial wastewater) were selected for ecotoxicity assessment. The 95% CI of the triple ecotoxicity tests for these toxicants demonstrated that the assessment platforms provided consistent results. These results indicate that the new ecotoxicological assessment is a universally applicable platform for measuring ecotoxicity. We propose that this method will be a platform for measuring the ecotoxicity of various hazardous substances including heavy metals.

CuSO_4_ and SDS were used to compare the 15% FBS/96 h and 1% FBS/6 h conditions. However, the concentrations of CuSO_4_ or SDS were different in each condition. For example, in the ecotoxicological assessment using CuSO_4_, concentrations of 0, 10, 50, 100, and 2000 ppm were used in the 15% FBS/96 h condition, whereas concentrations of 0, 6.25, 12.5, 25, 50, and 100 ppm were used in the 1% FBS/6 h condition. This inconsistency was due to the initial data performed to derive the EC_50_ of CuSO_4_ in the 15% FBS/96 h condition. The initial ecotoxicological assessment using the 15% FBS/96 h condition used CuSO_4_ at concentrations of 0, 1, 5, 10, 50, and 100 ppm. However, the EC_50_ could not be derived because the survival rate was still high even at 100 ppm (Figure S1A). Subsequent evaluations were performed using various concentrations, and the EC_50_ was finally derived at concentrations of 0, 10, 50, 100, 500, and 1000 ppm (Figure 3C). In addition, in the ecotoxicity evaluation using SDS, concentrations of 0, 200, 500, 1000, 2000, 4000, and 6000 ppm were used in the 15% FBS/96 h condition, and concentrations of 0, 12.5, 25, 50, 100, and 200 ppm were used in the 1% FBS/6 h condition. This inconsistency is also due to the initial data performed to derive the EC_50_ of SDS in the 15% FBS/96 h condition. In the initial ecotoxicity assessment using the 15% FBS/96 h condition, SDS at concentrations of 0, 1, 5, 10, 50, and 100 ppm were used. However, the survival rate was still high even at 100 ppm, so the EC_50_ could not be derived (Figure S1B). Subsequent evaluations were performed using various concentrations, and the EC_50_ was finally derived at concentrations of 0, 200, 500, 1000, 2000, 4000, and 6000 ppm (Figure 4C). In summary, the 15% FBS/96 h condition used higher concentrations of CuSO_4_ or SDS than the 1% FBS/6 h condition. This discrepancy could be due to the proliferation-inducing effect of the nutrients present in excess in the medium containing 15% FBS, which interfered with the cellular response to the toxicant [12,13]. In contrast, this study found that no cell proliferation or cell reduction occurred when cells were maintained in the medium containing 1% FBS for 6 h. These results suggest that there was no cell proliferation due to nutrient excess or cell reduction due to nutrient depletion during the 6 h period. Therefore, we applied the 1% FBS/6 h condition to the ecotoxicity assessment and were able to determine the EC_50_ at lower concentrations of CuSO_4_ or SDS.

## 5. Conclusions

In summary, we developed a novel platform to assess ecotoxicity using the 1% FBS/6 h treatment condition. To the best of our knowledge, this platform is the assessment tool with the shortest time required to obtain ecotoxicological data. Moreover, this platform provided more accurate ecotoxicity data than the traditional platform. Overall, our results will open a new paradigm in ecotoxicological assessment by providing an evaluation platform that enables rapid and accurate evaluation of toxicants.

## Figures and Tables

**Table 1:** Concentration ranges of toxic substances (metals and other substances) used in ecotoxicological assessments.

Toxicants	Concentration (ppm)					
CuSO_4_	0	10	50	100	2000	
CuSO_4_	0	6.25	12.5	25	50	100
SDS	0	200	500	1000	4000	6000
SDS	0	12.5	25	50	100	200
Titanium	0	6.25	12.5	25	50	100
Titanium nitride	0	6.25	12.5	25	50	100
Metolachlor	0	62.5	125	250	500	1000
Linuron	0	62.5	125	250	500	1000
2,4,6-Trichlorophenol	0	62.5	125	250	500	1000
Perfluorononanoic acid	0	62.5	125	250	500	1000

## Data Availability

Data are contained within the article and supplementary materials.

## Supplementary Materials

The following supporting information can be downloaded at: https://www.mdpi.com/article/10.3390/life14091119/s1, Table S1. Raw data for *C. carpio* cell proliferation in medium containing 15% FBS. Table S2. Raw data for *C. carpio* cell proliferation in medium containing 1% FBS. Table S3. Raw data for ecotoxicological assessment of CuSO_4_ (15% FBS/96 h). Table S4. Raw data for ecotoxicological assessment of CuSO_4_ (1% FBS/6 h). Table S5. Raw data for ecotoxicological assessment of SDS (15% FBS/96 h). Table S6. Raw data for ecotoxicological assessment of SDS (1% FBS/6 h). Table S7. Raw data for ecotoxicological assessment of titanium (1% FBS/6 h). Table S8. Raw data for ecotoxicological assessment of titanium nitride (TiN) (1% FBS/6 h). Table S9. Raw data for ecotoxicological assessment of metolachlor (1% FBS/6 h). Table S10. Raw data for ecotoxicological assessment of linuron (1% FBS/6 h). Table S11. Raw data for ecotoxicological assessment of 2,4,6-Trichlorophenol (1% FBS/6 h). Table S12. Raw data for ecotoxicological assessment of perfluorononanoic acid (PFNA) (1% FBS/6 h). Figure S1. Initial data on the ecotoxicity of CuSO_4_ and SDS in the 15% FBS/96 h condition.
