# Supplementary material for: Rapid and Accurate Ecotoxicological Assessment of Heavy Metals Using Cyprinus carpio Cells
Source: Life (Basel). 2024 Sep 5;14(9):1119. doi: 10.3390/life14091119 (PMC11432982; doi:10.3390/life14091119)
Supplement: Supplementary file 1 [file life-14-01119-s001.zip › life-3145732-supplementary.pdf]

Table S1. Raw data for *C. carpio* cell proliferation in medium containing 15% FBS.

Incubation time (h)	Raw data (Normalized with blank value)					
0	2891	2875	2875	2871	2829	2813
6	6152	6130	5940	5802	5643	5609
12	6020	6018	6000	5956	5940	5812
24	7977	7965	7743	7650	7534	7440
48	9129	9053	8955	8856	8806	8682
72	9454	9257	9067	9057	9053	9035
96	9085	8989	8878	8858	8734	8724

Table S2. Raw data for *C. carpio* cell proliferation in medium containing 1% FBS.

Incubation time (h)	Raw data (Normalized with blank value)					
0	2815	2757	2737	2713	2676	2662
6	2690	2666	2644	2636	2576	2574
12	2554	2540	2443	2393	2291	2235
24	2549	2541	2491	2487	2403	2343
48	2425	2359	2359	2347	2329	2295
72	2416	2214	2202	2144	2136	2100
96	2281	2197	2109	2093	2061	2033

Table S3. Raw data for ecotoxicological assessment of $CuSO_4$ (15% FBS/96 h).

Concentration (ppm)	Raw data (Normalized with blank value)								
Biological Repeats	# 1			# 2			# 3		
0	14766	14307	14675	14516	15703	15472	15735	15836	16727
10	9254	9198	13932	9287	8419	10018	10011	8862	10417
50	7324	8988	8169	8350	7044	7697	7462	8574	11687
100	8767	5835	5544	5531	5600	5802	6701	7767	6970
2000	6463	9122	6867	3830	4265	3602	4696	4595	3571

Table S4. Raw data for ecotoxicological assessment of $CuSO_4$ (1% FBS/6 h).

Concentration (ppm)	Raw data (Normalized with blank value)								
Biological Repeats	# 1			# 2			# 3		
0	2580	2434	2310	2947	2506	2306	2274	2478	2382
6.25	1868	1792	1842	1912	2124	2040	1784	1662	1552
12.5	1844	1982	1930	1908	1788	1890	1916	1900	1792
25	1866	1626	1488	1544	1718	1698	1642	1696	1840
50	1528	1732	1444	1552	1318	1648	1390	1420	1710
100	1096	832	718	986	692	828	792	866	970

Table S5. Raw data for ecotoxicological assessment of SDS (15% FBS/96 h).

Concentration (ppm)	Raw data (Normalized with blank value)								
Biological Repeats	# 1			# 2			# 3		
0	14757	14008	14449	13826	15004	14361	14109	14042	14419
200	8341	8419	8183	8705	8693	8697	8891	7826	8207
500	8149	7314	7352	8019	8361	8139	8729	8289	7862
1000	8713	7826	7128	6795	7162	7210	7118	7340	10908
2000	6115	5963	5765	5704	5949	6013	5855	6243	4902
4000	3891	4259	3847	4071	4157	4059	5678	4095	4203
6000	3323	3191	3265	3389	3221	3147	3255	3083	3141

Table S6. Raw data for ecotoxicological assessment of SDS (1% FBS/6 h).

Concentration (ppm)	Raw data (Normalized with blank value)								
Biological Repeats	# 1			# 2			# 3		
0	392	392	462	262	286	232	258	154	252
12.5	524	424	510	374	332	252	308	260	340
25	820	846	816	682	748	542	800	558	470
50	4804	5314	4552	4700	4321	4326	4500	5158	4570
100	5678	6071	4996	5338	5354	5719	4762	5662	5228
200	4924	4914	5086	6519	4978	4622	4896	4826	5999

Table S7. Raw data for ecotoxicological assessment of titanium (1% FBS/6 h).

Concentration (ppm)	Raw data (Normalized with blank value)								
Biological Repeats	# 1			# 2			# 3		
0	3127	3491	3323	4049	3535	4158	4060	3834	4322
6.25	3045	3395	3397	5452	3423	3527	3411	3681	3961
12.5	3051	3189	3529	3529	3211	3857	3343	3599	3907
25	1945	2281	2723	2707	2773	2771	2627	2917	3321
50	698	774	900	746	758	1126	1308	1604	1784
100	230	38	290	542	-180	100	-78	-138	242

Table S8. Raw data for ecotoxicological assessment of titanium nitride (TiN) (1% FBS/6 h).

Concentration (ppm)	Raw data (Normalized with blank value)								
Biological Repeats	# 1			# 2			# 3		
0	3003	3423	3773	3873	3503	4035	3791	4332	3717
6.25	2943	3765	3819	3767	3887	3971	4256	4410	3313
12.5	3209	3411	3579	3723	3817	3827	4121	4011	3827
25	1791	2289	2501	2583	2633	2791	2661	2437	2633
50	830	1432	1618	1350	1468	1756	1820	1620	1686
100	700	722	868	910	1216	1068	1116	894	1326

Table S9. Raw data for ecotoxicological assessment of metolachlor (1% FBS/6 h).

Concentration (ppm)	Raw data (Normalized with blank value)								
Biological Repeats	# 1			# 2			# 3		
0	1724	1726	1764	1908	1658	1914	2018	1788	1714
62.5	1741	1751	1867	1651	1677	1809	1845	1597	1765
125	1777	1705	1799	1707	1519	1571	1667	1631	1693
250	1567	1305	1223	1365	1145	1651	1269	1173	1471
500	1148	1176	1454	1024	988	948	1050	1016	994
1000	548	608	664	678	688	746	838	730	790

Table S10. Raw data for ecotoxicological assessment of linuron (1% FBS/6 h).

Concentration (ppm)	Raw data (Normalized with blank value)								
Biological Repeats	# 1			# 2			# 3		
0	2415	2457	2515	2603	2730	3178	2846	2792	2451
62.5	3007	2340	2408	2442	3181	2526	2769	2759	2094
125	2376	2254	2334	2104	2144	2486	2649	2609	2372
250	1814	1572	1808	2310	2094	1986	1790	1994	1500
500	1302	1216	1592	1390	1476	1070	1524	1414	1336
1000	608	700	900	776	854	812	612	576	596

Table S11. Raw data for ecotoxicological assessment of 2,4,6-Trichlorophenol (1% FBS/6 h).

Concentration (ppm)	Raw data (Normalized with blank value)								
Biological Repeats	# 1			# 2			# 3		
0	4577	3216	3658	3586	3532	3018	3132	3404	3466
62.5	3775	3436	3692	3837	3969	3632	3468	2982	2800
125	4404	4464	4182	4414	4188	4500	3959	4256	3535
250	3662	3644	3696	3777	3811	3584	3598	3152	3280
500	2628	3106	3108	2672	2860	2180	2478	3010	2136
1000	1371	1073	1754	1167	1417	1221	979	1089	765

Table S12. Raw data for ecotoxicological assessment of perfluorononanoic acid (PFNA) (1% FBS/6 h).

Concentration (ppm)	Raw data (Normalized with blank value)								
Biological Repeats	# 1			# 2			# 3		
0	4450	4330	4885	4853	5115	4645	5023	4817	4907
62.5	3162	3248	3226	3718	3504	3646	3584	3508	3454
125	1890	2092	2204	2392	2318	2322	2398	2464	2548
250	1800	1974	1726	2272	2198	2268	2128	2310	2330
500	1968	1882	2026	2170	2190	2290	2168	2170	2244
1000	1428	1672	1576	1942	1914	1888	1910	1924	1974

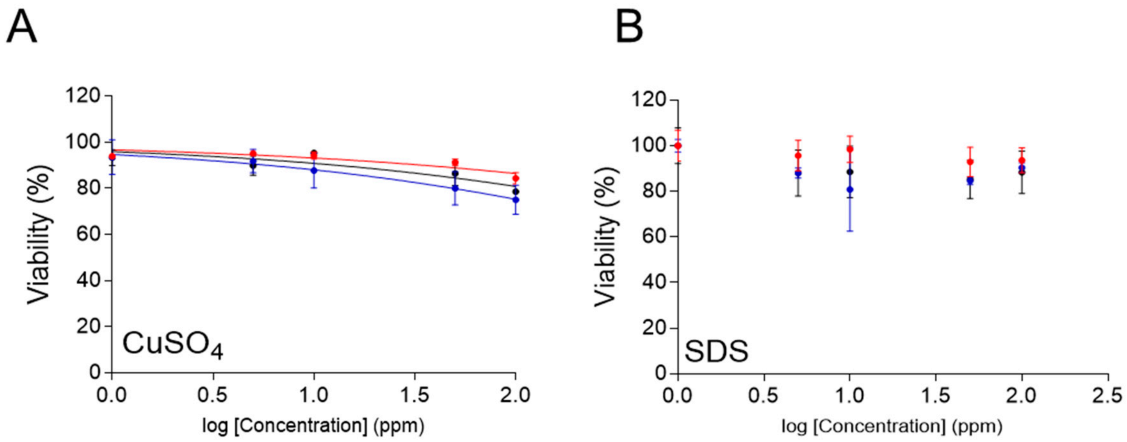

Figure S1. Initial data on the ecotoxicity of $CuSO_4$ and SDS in the 15% FBS/96 h condition. (A) In ecotoxicological assessment using medium containing 15% FBS, cell viability was assessed after treating *C. carpio* cells with different concentrations of $CuSO_4$ (0, 1, 5, 10, 50, and 100 ppm) for 96 h. All data presented were performed in biological triplicates using three samples in each experiment. (B) In ecotoxicological assessment using medium containing 15% FBS, cell viability was assessed after treating *C. carpio* cells with different concentrations of SDS (0, 1, 5, 10, 50, and 100 ppm) for 96 h. All data presented were performed in biological triplicates using three samples in each experiment.
